# High replacement of fishmeal by *Chlorella* meal affects intestinal microbiota and the potential metabolic function in largemouth bass (*Micropterus salmoides*)

**DOI:** 10.3389/fmicb.2022.1016662

**Published:** 2022-09-23

**Authors:** Zhimin Zhang, Longwei Xi, Haokun Liu, Junyan Jin, Yunxia Yang, Xiaoming Zhu, Dong Han, Shouqi Xie

**Affiliations:** ^1^State Key Laboratory of Freshwater Ecology and Biotechnology, Institute of Hydrobiology, Chinese Academy of Sciences, Wuhan, China; ^2^College of Advanced Agricultural Sciences, University of Chinese Academy of Sciences, Beijing, China; ^3^Hubei Engineering Research Center for Aquatic Animal Nutrition and Feed, Wuhan, China; ^4^The Innovative Academy of Seed Design, Chinese Academy of Sciences, Wuhan, China

**Keywords:** microalgae, fishmeal replacement, plant protein source, bacterial community, microbial function

## Abstract

Microalgae have beneficial effects on the performance of fish as additives and they are becoming a promising alternative to fishmeal as macronutrient ingredients. However, the impact on the fish intestinal microbiome and the function, caused by microalgae as protein sources in diets, remains unclear. This study aimed to determine the composition and potential function of the intestinal microbial community of largemouth bass (*Micropterus salmoides*) fed diets at five replacement levels (0, 25, 50, 75 and 100%) of fishmeal by *Chlorella* meal in a basal diet (400 g kg^−1^) after 8 weeks. The results showed significant decreases in unique amplicon sequence variants in the intestine at the higher levels of fishmeal replacement. At 50% of fishmeal replacement, dietary inclusions of *Chlorella* meal had no impact on species richness and Shannon diversity and the community structure of the intestinal microbiota. However, high levels of fishmeal replacement (75 and 100%) significantly induced intestinal community disturbance and diversity loss in largemouth bass. Responding to the high fishmeal replacement level, the dominant genus *Cetobacterium* and *Pleslomonas* sharply increased and several taxa from *Lactobacillus* decreased significantly. Functional data predicted by PICRUSt revealed that nutrition-related metabolism was dominant in the intestinal microbiota of fish fed all the five diets, although some potential functions, particularly amino acid and lipid metabolisms, and energy metabolism, were upregulated firstly, and then downregulated in fish fed diets with the increase of dietary *Chlorella* meal. Meanwhile, certain pathways were not enriched in intestinal microbiome until up to 75% of fishmeal replacement, such as carbohydrate metabolism, and cofactors and vitamins metabolism. To conclude, this study reveals that fishmeal replacement (50%) by *Chlorella* meal at the level of 237 g kg^−1^ in diets is feasible for largemouth bass without impairing the microbiome structure and the metabolism function, providing an alternative strategy for evaluating the possibility of fishmeal replacement by microalgae in aquafeeds.

## Introduction

Resilient and sustainable ingredients have to be used in aquafeeds as an alternative to traditional fisheries products for supporting the blue economy growth of aquaculture. Plant-based protein sources compose the major substitute of fishmeal for commonly farmed fish (Kaushik and Hemre, 2008). They are widely used in aquafeed and have been attractive alternatives to fishmeal (Tacon and Metian, 2008). It is possible to replace fishmeal partly or totally with soybean meal, rapeseed meal or other terrestrial protein sources for farmed species such as carps and tilapia, without compromising their growth (Anderson et al., 2016; Zhao et al., 2021). However, high levels of dietary plant protein inclusion may adversely affect the utilization and availability of feed compositions such as amino acids and minerals and therefore lead to fish growth retardation, disease resistance and gut health problems due to low palatability and occurrence of anti-nutritional factors in terrestrial meals (Francis et al., 2001; Li et al., 2009). This largely relies on the dietary inclusion levels, sources and fish species (Tacon and Metian, 2008; Ye et al., 2019; Abdel-Latif et al., 2022).

Fishmeal replacement with plant-based protein sources for carnivorous fish needs more studies because of higher protein requirements and lower plant protein inclusion compared to herbivorous and omnivorous fish (Tacon and Metian, 2008). In recent years, a universal increasing trend has been observed in the production of farmed fish, in particular carnivorous species such as largemouth bass (*Micropterus salmoides*) and yellow catfish (*Pelteobagrus fulvidraco*) in China (Fisheries Bureau of the Ministry of Agriculture and Rural Affairs, 2021), implying more demand for fishmeal or alternative protein sources in feed production (Oliva-Teles et al., 2015; Perez-Velazquez et al., 2019; Li et al., 2021a).

*Chlorella* as an important microalgae dietary ingredient source has good nutritional compositions and promotes farmed fish health (Alagawany et al., 2021). It has been incorporated into fish diets as feed additives for positive effects on feed utilization, growth, stress response and disease resistance (Alagawany et al., 2021). Previous studies indicated that the optimal levels of dietary *Chlorella* inclusion were lower than 5% for juvenile Korean rockfish *Sebastes schlegeli* (Bai et al., 2001), juvenile Japanese flounder *Paralichthys olivaceus* (Kim et al., 2002), rainbow trout fingerlings *Oncorhynchus mykiss* (Arteaga Quico et al., 2021), and juvenile crucian carp *Carassius auratus* (Luo et al., 2018). Moreover, the supplementation of microalgae can eliminate the detrimental effects of other plant protein substitutes (Bravo-Tello et al., 2017). Recently, the remarkable performance of microalgae as new protein sources for various aquatic animals has been assessed on its suitability in a growing number of studies. Rahimnejad et al. (2017) found dietary inclusion of 10–15% *Chlorella vulgaris* can positively affect antioxidant enzymes activity and lipid metabolism and promote the growth performance of olive flounder *P. olivaceus*. Notably, fishmeal replacement by *Chlorella* is as high as 100% for some aquatic species, without adverse effects (Pakravan et al., 2018).

For largemouth bass, *M. salmoides,* replacing fishmeal with alternative protein sources and/or improving the utilization was the object of most studies (Sampaio-Oliveira and Cyrina, 2008; Ding et al., 2015; He et al., 2020; Liu et al., 2021; Li et al., 2021b; Xu et al., 2022); however, few studies involved the use of microalgae such as *Chlorella* meal. Recently, a sister study revealed that fishmeal replacement with *Chlorella* meal can enhance the growth of largemouth bass and yellowness of fish body and dorsal muscle (Xi et al., 2022). However, 75% of total replacement of fishmeal by *Chlorella* meal in a basal diet led to lower feed efficiency and undesirable effects on fish health, suggesting that high fishmeal replacement by *Chlorella* meal could alter nutritional utilization and metabolism in the fish. It has been demonstrated that dietary change affects gut microbiota associated with host animals’ health such as nutrition and immunity function (Kononova et al., 2019; Rackerby et al., 2020; Wang et al., 2020). A review by Sagaram et al. (2021) mainly summarized recent developments on the role of microalgae as feed ingredients in the growth and immunomodulation of aquatic species as well as the gut microbiota. Only a very small minority of studies underlined an understanding of the intestinal microbiome of fish receiving microalgae diets and suggested the promotion of beneficial gut microbiota (Sagaram et al., 2021).

To attenuate tough competition for human food caused by the growth of protein demand in aquaculture, an accelerated paradigm shift has occurred towards new protein sources such as microalgae due to the potential of sustainable alternatives. The use of microalgae as bulk ingredients to replace fishmeal in several commercial species is growing significantly (Cao et al., 2018; Raji et al., 2018; Van Vo et al., 2020; Rosenau et al., 2021); however, few studies attempt to understand the effects of high fishmeal replacement with microalgae on gut microbiota and their role in fish nutrition and metabolisms. Based on recent evidence of the functional importance of gut microbiota in host animals (Rowland et al., 2018; Yukgehnaish et al., 2020; Mims et al., 2021), this study aimed to evaluate the effects of fishmeal replacement by *Chlorella* meal in diets on the gut microbiota of largemouth bass and further explore changes in the metabolic function of the microbiome to provide references of dietary *Chlorella* meal incorporation for fishmeal replacement.

## Materials and methods

### Experimental fish and facilities

Largemouth bass from the same batch was purchased from a commercial fish farm (Ezhou, Hubei Province, China). The fish were transferred into fiberglass cylinders and fed a commercial feed (>49% protein, Largemouth bass 1^#^) from Wuhan CP Aquatic Co., Ltd., Wuhan, Hubei, China for 2 weeks of environmental acclimation, and then a total of 300 largemouth bass (mean weight 17.64 ± 0.03 g) were randomly stocked to 15 circular 300-L tanks (five treatments with three replicates) with 20 fish per tank in a recirculation flow-through water system. During the experimental period, the water temperature was 28 ± 2°C and the photoperiod was maintained on a 12:12 light cycle. A piping system provided aeration for each tank through a blower, with >5 mg L^−1^ and a pump system distributed the water to each experimental tank. This experiment was performed at the Institute of hydrobiology, Chinese Sciences of Academy.

### Experimental diets

Five diets formulated to be isoproteic (50% crude protein) contained different graded levels of *Chlorella* meal in replacement of fishmeal in the basal diet (400 g kg^−1^) on a weight-for-weight basis. In this study, 0, 25, 50, 75, and 100% of fishmeal replacement were designed in the five experimental diets (FM100, FM75, FM50, FM25, and FM0), respectively. Fishmeal (Superprime, TASA Fish Product Co., Ltd., Peru) and *Chlorella* meal (Demeter Bio-Tech Co., Ltd., Wuhan, China) was used as the main protein sources in the diets. The formulation and proximate composition of the five experimental diets were shown in Table 1. A constant mixture of both plant and animal protein sources provided the remanent diet protein, and fish oil and soybean oil were used as lipid sources. The macro-ingredients were finely ground into fine powder. The micro-components, such as vitamin and mineral premixes were mixed thoroughly by the progressive enlargement method. All the ingredients were well mixed and pelleted in a laboratory pellet mill through a 3 mm die in Feed Research Institute, the Chinese Academy of Agricultural Sciences (Beijing, China). The dried diets were sieved and stored at 4°C in sealed plastic bags until they were used in the feeding trial.

**Table 1:** Formulation and proximate analysis (g kg^−1^, dry matter basis) of the experimental diets. FM100 represents the fishmeal diet without *Chlorella* meal; FM75, FM50, FM25, and FM0 represent the fishmeal diet with 25, 50, 75, and 100% fishmeal replacement by *Chlorella* meal, respectively.

Ingredients (g kg^−1^)	Diets				
	FM100	FM75	FM50	FM25	FM0
Fish meal	400	300	200	100	0
*Chlorella* meal	0	118.5	237	356	474.5
Soybean protein concentrate	130	130	130	130	130
Soybean meal	100	100	100	100	100
Gluten	50	50	50	50	50
Blood meal	40	40	40	40	40
Cassava starch	110	110	110	110	110
Fish oil	35	33.5	31.9	30.4	28.8
Soybean oil	35	33.5	31.9	30.4	28.8
Vitamin and mineral additives^#^	10	10	10	10	10
Monocalcium phosphate	15	15	15	15	15
Choline chloride	1	1	1	1	1
Microcrystalline cellulose	74	58.5	43.2	27.2	11.9
Proximate composition (%)					
Crude protein	49.93	50.10	50.30	50.16	49.64
Crude lipid	12.66	12.72	12.96	11.44	11.32
Gross energy (kJ g^−1^)	20.63	20.99	21.16	21.57	21.81

# Vitamin and mineral additives: from Guangdong Nutriera Group, Guangzhou, China. Vitamin additives, mg kg^−1^ diet: vitamin A 10; vitamin B_1_ 6; vitamin B_2_ 5; vitamin B_6_ 7.5; vitamin B_12_ (1%) 4; niacinamide 50; ascorbyl calcium phosphate (35%) 500; calcium pantothenate 20; biotin (2%) 2.5; folic acid 5; vitamin E (50%) 200; vitamin K_3_ 10; vitamin D_3_ 5; inositol 100; corn protein powder 75. Mineral additives, mg kg^−1^ diet: CuSO_4_·5H_2_O 10; FeSO_4_·H_2_O 300; ZnSO_4_·H_2_O 200; MnSO_4_·H_2_O 100; KIO_3_ (10%) 80; Na_2_SeO_3_ (10% Se) 67; CoCl_2_·6H_2_O (10% Co) 5; NaCl 100; zeolite 638.

### Feeding and sampling

Five diets were arbitrarily allocated into the 15 tanks with largemouth bass. The fish were fed twice daily at 8:30 and 16:30 to satiety for 8 weeks. The fish for sample collection were anesthetized with about 10 mg L^−1^ MS-222 (Sigma, St Louis, MO, United States). Nine faecal samples were collected from the distal intestine of 9 fish for each treatment (3 fish per tank). Briefly, faeces were sampled by abdominal stripping and squeezing the faeces into 1.5-mL sterile tubes. The samples were immediately stored at −80°C until the next processing. The experimental procedures such as sample collection and animal handling were conducted according to the Guiding Principles for Care and Use of Laboratory Animals approved by the Animal Ethical and Welfare Committee of Institute of Hydrobiology, Chinese Academy of Science (IHB, CAS, Protocol No. 2016–018).

### DNA extraction and sequencing

Total genomic DNA was extracted from the faeces of 45 samples using QIAamp DNA Stool Mini Kit (Qiagen, Hilden, NRW, Germany) according to the manufacturer’s instructions. The DNA concentration was measured by NanoDrop Spectrophotometer (ND-2000, ThermoFisher Scientific, United States), followed by gel electrophoresis. All extracted DNA was kept in the elution buffer and stored at −80°C. The V4-V5 hypervariable region of bacterial 16S rRNA gene was amplified with the specific primers: 515F, 5′-GTGCCAGCMGCCGCGGTAA-3′ and 907R, 5′-CCGTCAATTCCTTTGAGTTT-3′, with a 12-base barcode unique to each sample. The amplification program is composed of a denaturation step at 95°C for 2 min, followed by elongation for 25 cycles at 95°C for 30 s, 55°C for 30 s and 72°C for 30 s, and a final extension at 72°C for 5 min. The PCR reactions were performed in triplicate, with 20 μl of reaction system containing 4 μl of 5 × FastPfu Buffer, 2 μl of 2.5 mM dNTPs, 0.8 μl of each primer (5 μM), 0.4 μl of FastPfu Polymerase, and 10 ng of template DNA. The amplicons were extracted from 2% agarose gels and purified using the AxyPrep DNA GelExtraction Kit (Axygen Biosciences, Union City, CA, United States) according to the manufacturer’s protocols and quantified with QuantiFluor™-ST for the Qubit fluorometer (Promega, United States). An equimolar pool was prepared for amplicon library, which was subsequently used for paired-end sequencing (2 × 250) on an Illumina Hiseq2500 platform according to the standard protocols. Raw reads obtained in this study are deposited into the NCBI Sequence Read Archive (SRA) database with the accession number: PRJNA820974.

### Bioinformatic analyses

Data processing was analyzed using Quantitative Insights into Microbial Ecology version 2 (QIIME2) (Bolyen et al., 2019). Raw sequences were quality trimmed with an average quality score > 20. Paired-end reads were merged into full-length sequencing using FLASH v1.2.7 (Magoc and Salzberg, 2021). Chimeric sequences were identified and removed using UCHIME v4.2. Further, the DADA2 package was used to filter and truncate low-quality sequences (Callahan et al., 2016), and obtain clean amplicon sequence variant (ASV) sequences. The phylogenetic affiliation of each ASV sequence was taxonomically annotated by UCLUST v1.2.22q against the Silva (SSU132) 16S rRNA database.

### Diversity and statistical analysis

To control the inequality of sequencing depth, the raw data were normalized to the lowest sampling depth of about 30,000 sequences per sample. The α-diversity, including the Chao1, and Shannon diversity indices among intestinal microbial communities, was computed using Mothur (v.1.21.1), and the β-diversity was evaluated using QIIME 2. The significant α-diversity difference among groups was calculated by using a nonparametric Kruskal–Wallis test. The β-diversity was clustered by weighted and unweighted UniFrac distance metrics. The analysis of diversity was visualized by nonmetric multidimensional scaling (NMDS). Multivariate statistical analysis for the β-diversity was carried out using the PERMANOVA test to assess the effect of fishmeal replacement with *Chlorella* meal at different graded levels. Shared and unique ASVs among the treatments were presented in Venn diagrams. All the significance values were considered significantly different when *p* < 0.05.

## Results

### Sequencing data and ASV classification

**Figure 1:**
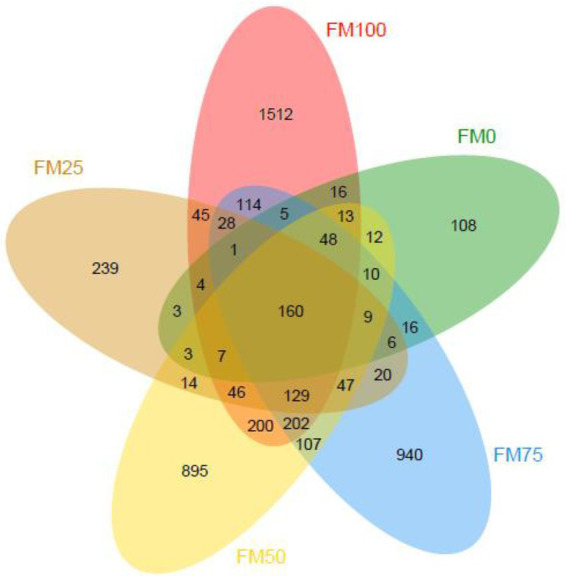
Venn diagram showing the observed ASVs of gut microbial communities from the five different levels of fishmeal replacement groups of largemouth bass. FM100 represents the fishmeal diet without *Chlorella* meal; FM75, FM50, FM25, and FM0 represent the fishmeal diet with 25, 50, 75, and 100% fishmeal replacement by *Chlorella* meal, respectively.

In this study, a total of 2,161,917 raw sequences from 45 gut samples of the largemouth bass were obtained and subsequently, 2,053,614 clean sequences (from 31,388 to 59,249) were classified into ASVs after quality filtering. Good’s coverage of all the gut samples was more than 0.999, suggesting that microbial phylotypes in the gut samples were well identified. The rarefaction curves of observed species indicated sufficient sequence data to produce stable and unbiased estimates of microbial species richness (data not shown). Figure 1 shows ASVs of gut microbial communities from largemouth bass fed the five different diets with fishmeal replacement by *Chlorella* meal. The averaged ASVs from the largemouth bass gut for FM100, FM75, FM50, FM25 and FM0 groups were 465, 305, 356, 120 and 77, respectively. The ASVs unique from the gut of largemouth bass in FM100, FM75, FM50, FM25 and FM0 groups were 1,512, 940, 895, 239 and 108, respectively; and the shared ASVs among the five groups were 160.

### Changes in alpha diversity of gut microbiota from largemouth bass

**Figure 2:**
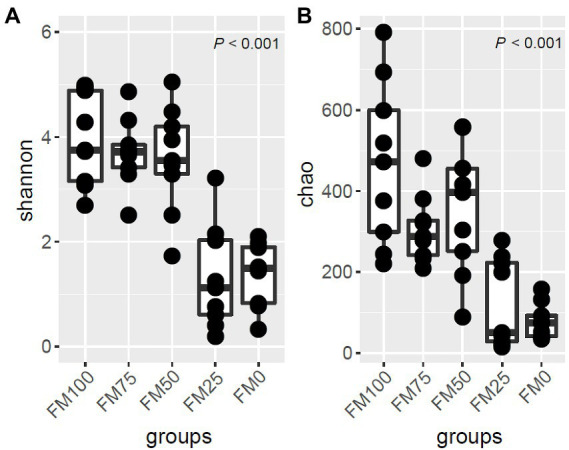
The alpha-diversity and ASV distribution of gut microbial community in largemouth bass fed with different levels of *Chlorella* meal diets. **(A)** Box plot showing the alpha diversity indices and **(B)** Venn diagram showing the shared ASVs and unique ASVs in the five treatments. FM100 represents the fishmeal diet without *Chlorella* meal; FM75, FM50, FM25 and FM0 represent the fishmeal feed with 25, 50, 75 and 100% fishmeal replacement by *Chlorella* meal, respectively.

Shannon index and Chao1 were used to calculate the alpha diversity of largemouth bass among the five groups using the rarefaction ASV sets (Figure 2). The Shannon diversity of the five groups was significantly different (*p* < 0.01). The Shannon diversity of FM50 and FM75 was similar to that of FM100. The three groups had significantly higher diversities than FM0 and FM25. Similarly, the Chao1 of the five groups differed significantly (*p* < 0.01), with higher species richness in FM100, FM75 and FM50 groups than in FM25 and FM0 groups (Figure 2). The results suggest that < 50% of fishmeal replacement have no impact on the gut microbial diversity and richness of largemouth bass, while the higher replacement decreases the alpha diversity.

### Differences in taxonomic composition of gut microbiota from largemouth bass

**Figure 3:**
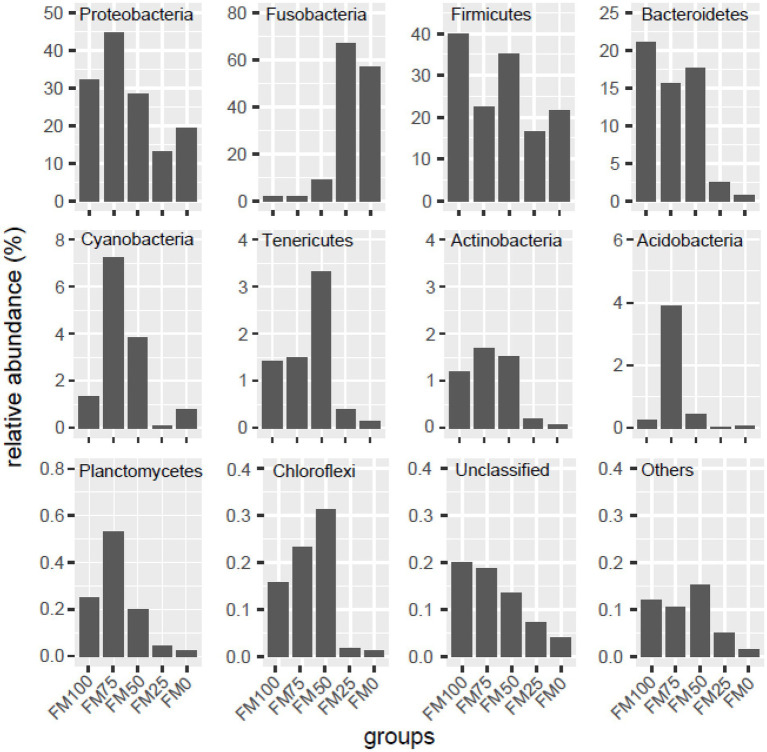
Taxonomic distribution of gut microbial community at the phylum levels among the five groups of largemouth bass. Unclassified, the unclassified phyla; others, the other known phyla. FM100 represents the fishmeal diet without *Chlorella* meal; FM75, FM50, FM25, and FM0 represent the fishmeal diet with 25, 50, 75, and 100% fishmeal replacement by *Chlorella* meal, respectively.

In this study, compositional differences were observed in the gut microbiota of largemouth bass among the five groups at the phylum levels. We listed the ten top most abundant phyla, while unclassified phyla were named “Unclassified” and the remaining phyla further were merged “Others,” as shown in Figure 3. The abundant phyla from the five groups of largemouth bass were Proteobacteria, Firmicutes, Fusobacteria, Bacteroidetes, followed by Cyanobacteria, Tenericutes, Actinobacteria, Planctomycetes, Acidobacteria and Chloroflexi. However, the abundance of the dominant microbial taxa was significantly different among the five groups (Figure 3). In the FM100 group, Firmicutes (39.8%), Proteobacteria (32.1%), Bacteroidetes (21.1%) were the three most dominant phyla. In the FM75 group, Proteobacteria was the most abundant taxon (44.7%), followed by Firmicutes (22.44%), Bacteroidetes (15.6%) and Cyanobacteria (7.2%). The four phyla dominated in the FM50 group and their relative abundance was 28.4, 35.2, 17.7 and 3.8%, respectively. In FM25 and FM0 groups, Fusobacteria was the dominant microbial taxon (66.9 and 57.1%), followed by Firmicutes (16.7 and 21.7%) and Proteobacteria (13.2 and 19.3%). Proteobacteria, Cyanobacteria, Actinobacteria, Tenericutes, Acidobacteria and Chloroflexi firstly increased, and then decreased in the gut of largemouth bass fed diets with increases of fishmeal replacement by *Chlorella* meal. The abundance of Fusobacteria significantly increased at up to 75% of fishmeal replacement, while those of Firmicutes, Bacteroidetes, “Unclassified” and “Others” decreased (Figure 3).

**Figure 4:**
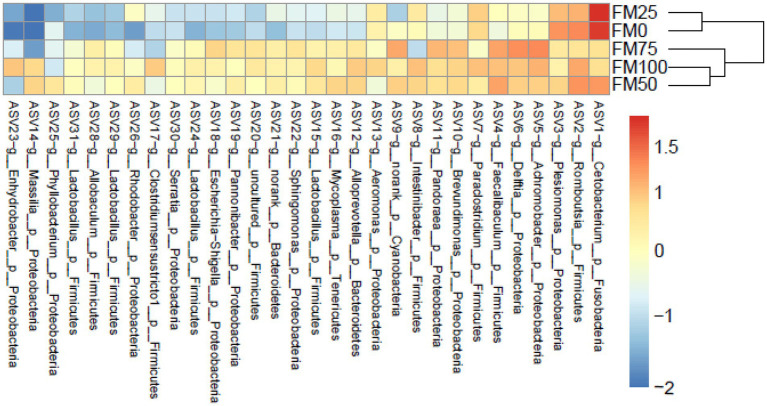
Heatmap showing the relative abundance reveals the compositional differences of the gut microbiota of largemouth bass fed with different levels of *Chlorella* meal diets. Bray-Curtis clustering based on the top 30 ASVs in the gut microbiota. FM100 represents the fishmeal diet without *Chlorella* meal; FM75, FM50, FM25, and FM0 represent the fishmeal diet with 25, 50, 75, and 100% fishmeal replacement by *Chlorella* meal, respectively.

Based on the ASVs level, we further found significant microbial differences among the five groups based on the top 30 ASVs by heatmap analysis (Figure 4). ASV1 (belonging to genus *Cetobacterium*) and ASV3 (*Pleslomonas*) were greatly enriched in FM25 and FM0 groups compared to other groups; meanwhile, the abundant ASV5 (*Achromobacter*), ASV4 (*Faecalibaculum*) and ASV6 (*Delftia*) decreased significantly. In addition, several ASVs such as ASV15, ASV24, ASV29 and ASV31 from the genus *Lactobacillus* greatly reduced the abundance in FM25 and FM0 groups.

### Beta diversity of gut microbial communities from largemouth bass

**Figure 5:**
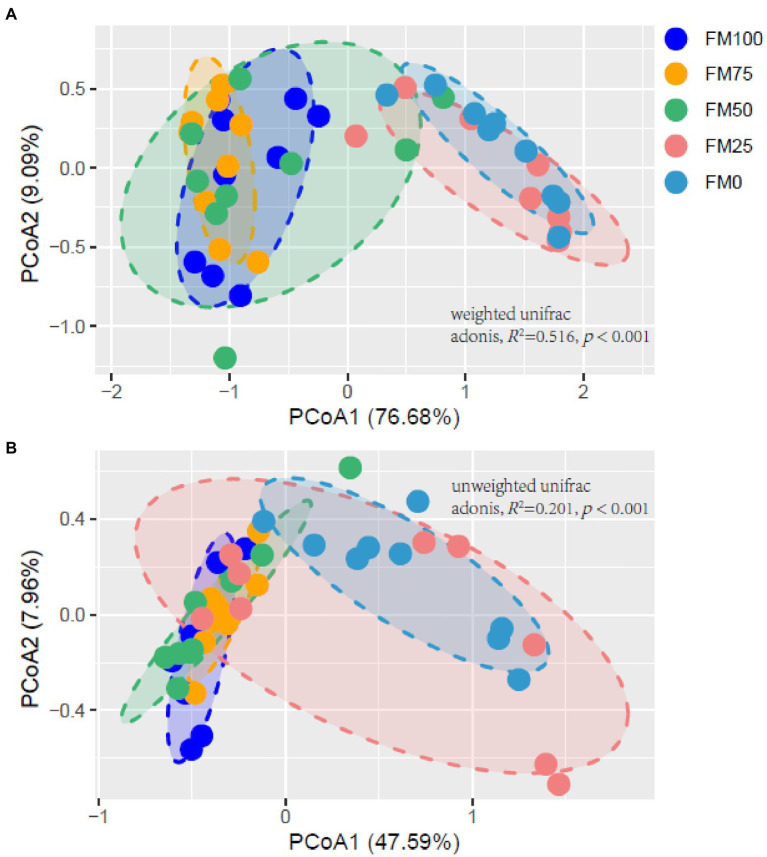
Principal Co-ordinate analysis diagrams **(A)** based on weighted unifrac distance and **(B)** based on unweighted unifrac distance showing the gut microbial distribution among the five groups of largemouth bass. FM100 represents the fishmeal diet without *Chlorella* meal; FM75, FM50, FM25, and FM0 represent the fishmeal diets with 25, 50, 75, and 100% fishmeal replacement by *Chlorella* meal, respectively.

Figure 5 shows the principal Co-ordinate analysis (PCoA) of beta diversity associated with the gut community variance of the five groups of largemouth bass based on Weighted (Figure 5A) and Unweighted (Figure 5B) Unifrac distances. The PCoA diagrams both showed that samples from the FM100 group closely overlapped those from FM75 and FM50 groups, but separated from those from FM25 and FM0 groups along the PC1 coordinate axis (Figure 5). Further, the two beta-diversity metrics were evaluated with PERMANOVA, indicating significant differences in gut microbial communities of largemouth bass fed with different levels of *Chlorella* meal diets (Weighted, *R*^2^ = 0.516, *p* < 0.001; Unweighted, *R*^2^ = 0.201, *p* < 0.001). Subsequently, we used the PERMANOVA pair-wise test to analyze the differences between groups (Table 2). As intestinal samples plotted in Figure 5, the communities had no significant difference between the FM75 and FM50 groups (Table 2, *p* > 0.05 for both metrics), and were similar to that of the FM100 group (*p* > 0.05 for both metrics). FM25 and FM0 groups had no community differences (*p* > 0.05); however, both of them were significantly different from the other three groups (*p* < 0.05 for both metrics).

**Table 2:** The PERMANOVA Pair-wise test for the gut microbial community from largemouth bass fed with different levels of *Chlorella* meal diets. Bold values represent statistically significant differences between groups. FM100 represents the fishmeal diet without *Chlorella* meal; FM75, FM50, FM25, and FM0 represent the fishmeal diet with 25, 50, 75, and 100% fishmeal replacement by *Chlorella* meal, respectively.

Adonis	Diets	FM100	FM75	FM50	FM25	FM0
Weighted unifrac, (value of *p*)	FM100	1				
	FM75	0.101	1			
	FM50	0.831	0.117	1		
	FM25	**0.001**	**0.001**	**0.001**	1	
	FM0	**0.001**	**0.001**	**0.001**	0.427	1
Unweighted unifrac, (value of *p*)	FM100	1				
	FM75	0.191	1			
	FM50	0.694	0.896	1		
	FM25	**0.002**	**0.016**	**0.012**	1	
	FM0	**0.001**	**0.001**	**0.001**	0.344	1

### Metabolic functions of gut microbial communities from largemouth bass

**Figure 6:**
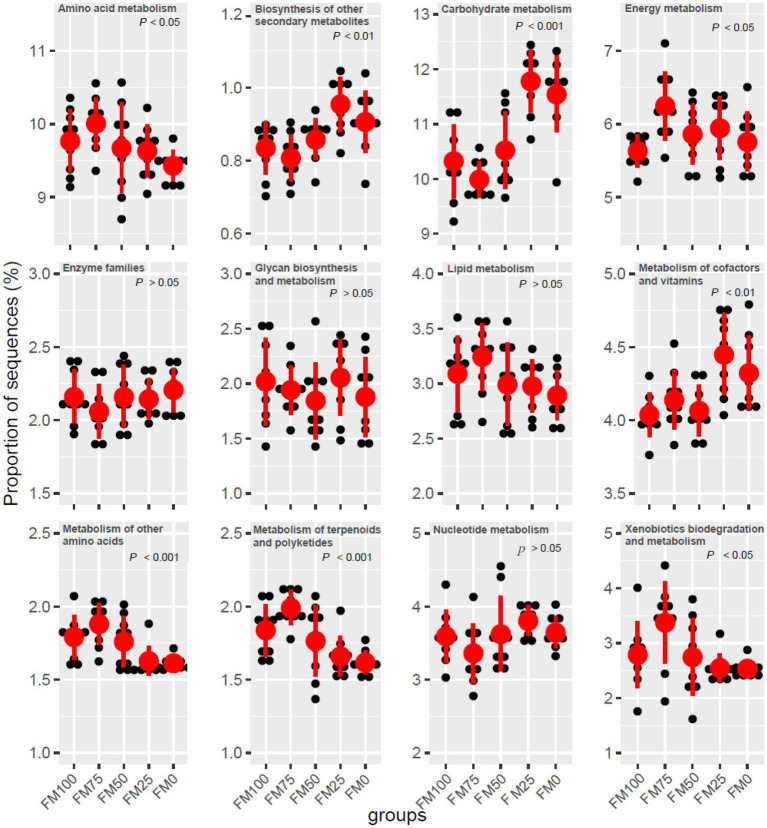
Functional metabolism analysis of KEGG pathways in gut microbial communities from the five groups of largemouth bass fed with different levels of *Chlorella* meal diets. FM100 represents the fishmeal diet without *Chlorella* meal; FM75, FM50, FM25, and FM0 represent the fishmeal feed with 25, 50, 75, and 100% fishmeal replacement by *Chlorella* meal, respectively.

Microbial functions from the five groups of the largemouth bass were predicted based on KEGG (Table 3). Seven functional categories were predicted in this study and the dominant predicted function involved Metabolism, followed by Genetic Information Processing and Environmental Information Processing. These functional categories were related to significant differences in gut microbial communities among the five groups, with fewer functional genes enriched in FM25 and FM0 groups (Table 3). Further functional categories from Metabolism also showed differences in metabolism-related pathways among the five groups (Figure 6). Compared to the FM100 group, the pathways of amino acid metabolism, energy metabolism, lipid metabolism, and metabolism of other amino acids, metabolism of terpenoids and polyketides, and xenobiotics biodegradation were more significantly enriched in FM75 group. Subsequently, these pathways showed a decrease in the FM50 group, meanwhile, lower enrichment was observed in FM25 and FM0 groups (Figure 6). On the contrary, the pathways of biosynthesis of other secondary metabolisms, carbohydrate metabolism, and metabolism of cofactors and vitamins were significantly more enriched in the two groups compared to FM100 group.

**Table 3:** KEGG function prediction of the gut microbial communities from largemouth bass fed with different levels of *Chlorella* meal diets. FM100 represents the fishmeal diet without *Chlorella* meal; FM75, FM50, FM25, and FM0 represent the fishmeal diet with 25, 50, 75, and 100% fishmeal replacement by *Chlorella* meal, respectively. The values represent the numbers of predicted functional genes of gut microbial communities. Different letters within the same row indicate significant differences.

KEGG function classification	FM100	FM75	FM50	FM25	FM0
Cellular processes	1567782 ± 704033^a^	1413728 ± 480186^a^	1570917 ± 809433^a^	782449 ± 555537^b^	1044642 ± 522318^ab^
Environmental information processing	5928248 ± 2150282^ab^	5607656 ± 1520981^ab^	6610498 ± 2664878^a^	3995865 ± 1806244^b^	4902084 ± 1857974^ab^
Genetic information processing	6718653 ± 1678032^a^	6099242 ± 1249280^ab^	7124538 ± 1724333^a^	4285034 ± 1581447^b^	4919105 ± 1621936^b^
Human diseases	378430 ± 96133^a^	405772 ± 115818^a^	407832 ± 157295^a^	202069 ± 91095^b^	249904 ± 92704^b^
Metabolism	18492114 ± 4955473^a^	1831142 ± 4621194^a^	19877153 ± 6176083^ab^	12600281 ± 4059650^c^	14407436 ± 4364602^dc^
Organismal systems	270076 ± 72973^a^	275435 ± 70329^a^	283076 ± 110871^a^	162386 ± 44359^b^	184282 ± 48191^b^
Unclassified	5424761 ± 1606937^a^	5169782 ± 1235362^a^	5725901 ± 1820580^a^	3703406 ± 1350150^b^	4341982 ± 1394304^ab^

## Discussion

It is a common practice to utilize microalgae as either a protein source ingredient to reduce the use of fishmeal without compromised performance and to achieve protein source sustainability, or to include it as a low-level dietary supplement to improve fish health (Bai et al., 2001; Perez-Velazquez et al., 2019; Van Vo et al., 2020). Our recent study reveals that partial fishmeal replacement by *Chlorella* meal in diets contributes to better growth (Xi et al., 2022). However, the effects of replacing fishmeal with *Chlorella* meal on fish gut microbiota remain to be explored. Here, we reported that, depending on the levels of fishmeal replacement by *Chlorella* meal, the effects of dietary *Chlorella* meal on the gut microbiome of largemouth bass are significantly different. Besides, based on metagenome predictions of the microbial community, we speculated that dietary inclusion of *Chlorella* meal can promote metabolic functions of the gut microbiome; however, the consequences of high fishmeal replacement on microbial metabolisms may be unfavourable for the growth of largemouth bass.

An undesirable effect of dietary compositional changes consists of a loss of microbial diversity and intestinal disturbance (Sonnenburg et al., 2016; Mims et al., 2021). Green et al. (2013) found that salmon fed diets with 5 and 10% of soybean protein concentrate inclusions increased the bacterial diversity of the intestine, but resulted in the presence of bacteria not normally associated with marine fish (*Escherichia* and *Propionibacterium*). Similarly, some studies reported that low-level dietary microalgae supplementation resulted in an increasing trend in microbial diversity (Lyons et al., 2017; Jorge et al., 2019; Nayak et al., 2020). In this study, dietary *Chlorella* meal had no impact on microbial diversity and richness species when the level of fishmeal replacement was below 50%, while higher levels of replacement led to a remarkable diversity loss, indicating different responses of the microbes in the gut to dietary inclusion levels. It can be speculated that fishmeal replacement alters original fermentable substrates and thus suppress the growth of certain bacteria within excessive inclusions of *Chlorella* meal groups. Gut microbial diversity is associated with feeding habits, decreasing from herbivorous to omnivorous to carnivorous species. This is related to the co-evolution of hosts and their diets. High fishmeal replacement diets (such as FM25 and FM0) were fed to carnivorous largemouth bass, suggesting the gut microbiota may be exposed to plant-based diets due to high inclusions of *Chlorella* meal. In addition, the microbial diversity loss could be considered harmful in that a significant reduction in microbial diversity may provide more competition for opportunistic or invading pathogens potentially entering the digestive tract of fish.

The present results revealed replacing fishmeal with *Chlorella* meal significantly affected the gut microbial compositions of largemouth bass. The dominant ASVs were classified into Proteobacteria and Firmicutes, followed by Bacteroidetes and Fusobacteria. Similar to the results reported in previous studies, Proteobacteria and Firmicutes not only dominate in the gut of largemouth bass (Zhou et al., 2021) but also in diverse aquatic animal species such as red drum (*Sciaenops ocellatus*) (Yamamoto et al., 2021), totoaba (*Totoaba macdonaldi*; Larios-Soriano et al., 2022), suggesting the advantage of these taxa inhabiting the gut of aquatic animals. It has been reported that some taxa from Proteobacteria can get carbon and energy source by using fatty acids and branched amino acids (Kazakov et al., 2009). The abundance of Proteobacteria increased with increasing dietary inclusion level of *Chlorella* meal from 0 to 25%, and then decreased significantly beyond 25% replacement level; meanwhile, the abundance of Firmicutes significantly decreased with increasing level of *Chlorella* meal. A similar trend is also found in pearl gentian grouper (♀*Epinephelus fuscoguttatus* × ♂*E. lanceolatu*) fed diets with high levels of cottonseed protein concentrate (Chen et al., 2020). In particular, the high fishmeal replacement by *Chlorella* meal lowered microbial taxa from *Lactobacillus* which are the fermenting organisms associated with the production of lactic acid and acetic acid from the fermentation of plant-based protein and energy sources. This suggests that high fishmeal replacement by plant protein could inhibit the two dominant taxa in carnivorous fish species. The low abundance of Fusobacteria existed in multiple fish species (Sullam et al., 2012); however, Zhang et al. (2017) revealed the prevalence and dominance of Fusobacteria in the fish gut, regardless of species and feeding habits. This indirectly reflects the importance of external factors including dietary compositions shaping this taxon. Our recent work also showed the enrichment of Fusobacteria in carnivorous and herbivorous fish species fed a high protein diet (Zhang et al., 2019). In the present study, the abundance of Fusobacteria in FM25 and FM0 groups (> 60%) sharply increased, compared to the control group (< 2%). It may be explained that it is more difficult to digest plant-based protein diets than fishmeal-based diets for largemouth bass (Skrede et al., 2011) so the enrichment of Fusobacteria would enhance the protein utilization. Indeed, Xie et al. (2021) recently reveals the fermentation product of *Cetobacterium somerae* belonging to Fusobacteria is beneficial for fish gut and liver health to improve the efficacy of fishmeal replacement by plant proteins. In addition, this genus can contribute to the degradation and metabolism of dietary vitamin B_12_ (Tsuchiya et al., 2008).

Regarding dietary effects of fishmeal replacement on gut microbial community, beta diversity analysis was used to visualize the community structure change of samples (Gatesoupe et al., 2014; Larios-Soriano et al., 2022). As we speculated, the microbial communities differed among the five groups. The gut samples from FM100, FM75 and FM50 groups overlapped closely but separated from those from FM25 and FM0 groups, indicating the significant effect on gut microbial community. Combined with previous results in largemouth bass (Xi et al., 2022) and other species on growth performance and feed utilization, this study further demonstrates the alterations of gut homeostasis and the potential negative effects caused by high proportions of plant protein inclusion. In some aquaculture species, partial substitutions of fishmeal by plant ingredients (such as soy protein concentrate) negatively affects gut morphology by decreasing mucosal fold width and shortening the microvilli in the gut of fish (Green et al., 2013; Larios-Soriano et al., 2022). Although the affected intestinal integrity and physiology could be attributed to fishmeal replacement, changes in gut structure could be also associated with the gut microbial alteration or dysbiosis, especially autochthonous microbiota, adhered to the intestinal epithelium (Kononova et al., 2019; Larios-Soriano et al., 2022).

The functional pathways expressed in five groups of largemouth bass fed different inclusions of *Chlorella* meal were primarily associated with metabolism. However, the numbers of gene expressions were significantly different among the groups. Some studies recently have reported the important metabolic function of gut microbiota in fish and other animals and the metabolic differences associated with plant protein sources (Kononova et al., 2019). In carnivorous fish, the high protein demand is largely attributed to the use of amino acids from dietary proteins in energy metabolism (Kaushik and Seiliez, 2010). The functional gene enrichment for energy metabolism was highest in the gut for largemouth bass fed 25% *Chlorella* meal in the FM75 group. It implies that dietary nutrients escaping digestion by endogenous digestive enzymes (such as trypsin or chymotrypsin) are better fermented and utilized by the distal intestinal bacteria in largemouth bass, potentially contributing to the optimal growth (Xi et al., 2022). In addition, the elevation of gene pathways dedicated to amino acid metabolism and lipid metabolism further indicates the gut microbiota involved in the fermentative processes. High fishmeal replacement can provoke metabolic disorders and growth retardation by reducing these gene pathways and triggering the enhancement of other metabolisms. The carbohydrate metabolic pathway expressed by the gut microbiota was significantly enriched in largemouth bass fed diets with more than 75% fishmeal replacement by *Chlorella* meal. It is likely responsible for decomposing extra carbohydrates from *Chlorella* meal in the diets (Skrede et al., 2011; Shah et al., 2018). The capability of carbohydrate metabolism in the gut microbiota of carnivorous fish is inferior to that of herbivorous and omnivorous fish. In addition, dietary carbohydrate sources and forms affect the intestinal microbiota and metabolic response of fish differently (Gatesoupe et al., 2014). In largemouth bass, 15% of dietary starch negatively affects gut microbiota and decreases the production of short-chain fatty acids such as acetate, propionate and butyrate in the gut (Zhou et al., 2021). As a whole, high fishmeal replacement protocols by microalgae should consider not only the contents of carbohydrates but also their sources and profiles from the microalgae inclusions in fish.

## Conclusion

The present study shows that up to 50% (200 g kg^−1^) of fishmeal could be replaced with *Chlorella* meal in the basal diet for largemouth bass juveniles without causing significant effects on the intestinal microbial community. Further replacement of fishmeal up to 75% or complete substitution leads to the alteration of gut microbial structure. In addition, microbial functional data obtained in this study demonstrate that replacing fishmeal significantly affects the capability of intestinal microbiota associated with the metabolism of dietary nutrients such as protein-related amino acids and lipids. Notably, 25% of fishmeal replacement enhances the metabolisms, as well as energy metabolism, and may therefore achieve improvement in dietary utilization and the performance of largemouth bass as reported in our previous study (Xi et al., 2022), further revealing the feasibility of partial substitution of fishmeal by *Chlorella* meal.

## Data availability statement

The datasets presented in this study can be found in online repositories. The names of the repository/repositories and accession number(s) can be found at: https://www.ncbi.nlm.nih.gov/, PRJNA820974.

## Ethics statement

The animal study was reviewed and approved by the Animal Ethical and Welfare Committeethe of Institute of Hydrobiology, Chinese Academy of Science.

## Author contributions

ZZ, DH, and SX designed the experiment. ZZ, LX, HL, JJ, and YY conducted the experiment. ZZ and LX analyzed the data. ZZ wrote the manuscript. DH, XZ, and SX revised the manuscript. All authors contributed to the article and approved the submitted version.

## Funding

This research was funded by the National Key Research and Development Program of China (2018YFD0900400), China Agriculture Research System of MOF and MARA (CARS-46), Fund Project in State Key Laboratory of Freshwater Ecology and Biotechnology (2019FBZ02, 2019FBZ05), and Hubei High-tech Innovation and Business Incubation Center (2019-02-055).

## Conflict of interest

The authors declare that the research was conducted in the absence of any commercial or financial relationships that could be construed as a potential conflict of interest.

## Publisher’s note

All claims expressed in this article are solely those of the authors and do not necessarily represent those of their affiliated organizations, or those of the publisher, the editors and the reviewers. Any product that may be evaluated in this article, or claim that may be made by its manufacturer, is not guaranteed or endorsed by the publisher.
